# Supplementary figures and images for: Locus coeruleus injury modulates ventral midbrain neuroinflammation during DSS-induced colitis
Source: bioRxiv. 2024 Feb 13:2024.02.12.580010. Preprint. [Version 1] doi: 10.1101/2024.02.12.580010 (PMC10888767; doi:10.1101/2024.02.12.580010)

**a** Sample yield metrics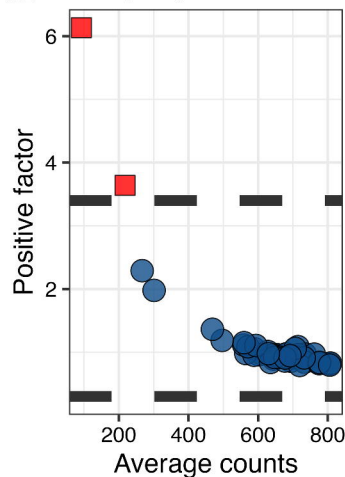**b** Imaging QC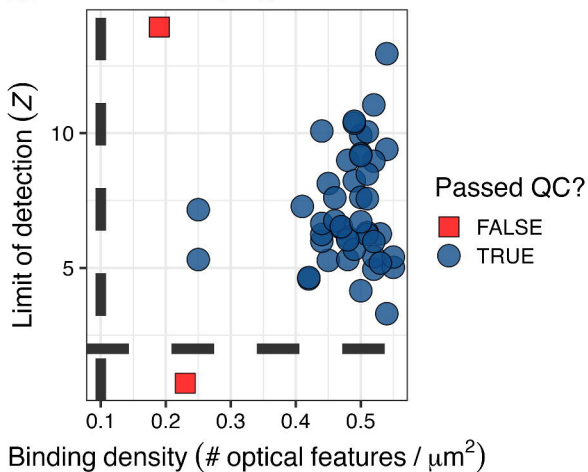**c**

Housekeeping gene counts

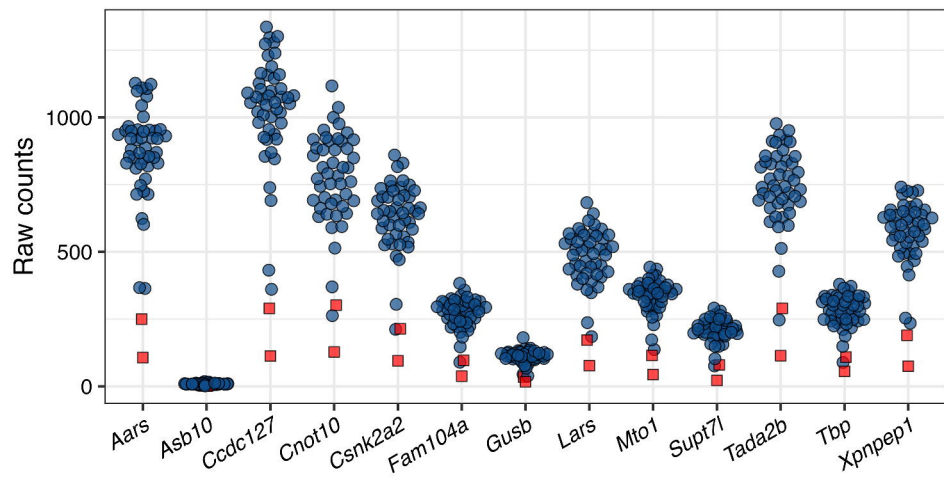

Supplement: 1 — Figure S1: Quality assessment of NanoString nCounter data. (a) Sample yield metrics, with the average transcript count for each sample on the x-axis and the positive library scaling factor based on the positive control probes on the y-axis. Dashed lines mark the lower and upper cutoffs for the positive factor. (b) Imaging quality metrics, with the binding density (expressed as the number of features per square micron) on the x-axis and the limit of detection on the y-axis. Dashed lines mark the lower bounds for each metric. (c) Raw counts of all housekeeping genes included in the panel. From this figure, Asb10 was omitted as a housekeeper due to its low counts overall. For each panel, each dot represents a sample, and samples are colored by whether they pass all QC checks and were thus carried into downstream analyses. [file NIHPP2024.02.12.580010V1-supplement-1.pdf]
